# Hands-Only Cardiopulmonary Resuscitation Education for Elementary School Students in Korea: Tracking by School Grade, Physical Characteristics, and Physical Strength

**DOI:** 10.3389/ijph.2023.1606054

**Published:** 2024-02-05

**Authors:** Jang Sik Ko, Seon Rye Kim, Byung Jun Cho

**Affiliations:** ^1^ Department of Paramedicine, Kangwon National University, Samcheok, Republic of Korea; ^2^ Department of Healthcare Management, Youngsan University, Yangsan, Republic of Korea

**Keywords:** cardiopulmonary resuscitation, physical strength, chest compression depth, schoolchildren, education

## Abstract

**Objectives:** This study aimed to assess variations in chest compression depth among Korean elementary school students based on grade, physical characteristics, and strength.

**Methods:** The study involved 140 children in the third to sixth grades from elementary schools. Before providing cardiopulmonary resuscitation (CPR) education, we assessed height, weight, BMI, grip strength, and back strength. Subsequently, CPR education was administered, followed by individual measurements of compression depth. The factors related to compression depths was analyzed using t-test, ANOVA and multivariable regression.

**Results:** The mean compression depth was consistently lower than the guideline standard across all grades, indicating grade-dependent differences (*p* = 0.000). Moreover, height, weight, BMI, grip strength and back strength increased, exhibited significant increases with grade (*p* = 0.000). In multivariable regression analysis, it was observed that as grade increased, chest compression depth increased by 0.701 cm (*p* = 0.000).

**Conclusion:** School grade significantly influenced achieving the proper chest compressions depth, no notable correlation found for physical factors. Thus, a strategy emphasizing the importance of sufficient chest compressions during CPR education, particularly targeting elementary school students, seems necessary to encourage greater effort.

## Introduction

The incidence of acute cardiac arrest in Korea increased from 2006 to 2015 at a rate of 58.6 cases per 100,000 and later decreased slightly in 2016 to 58.4 per 100,000 people; in fact, the incidence of acute cardiac arrest patients is increasing every year, higher than values of 37.2 for gastric cancer, 8.8 for transportation accidents, and 22.2 for bronchial and lung cancer [1]. The rate of CPR education for non-medical personnel was 1.9% in 2008, and it has risen every year to 23.5% in 2018, roughly 12 times its value a decade ago [2]. Despite these improvements, however, the rate of CPR education for non-medical personnel in Korea is still low compared to 33.3% in the United States and 34.8% in Japan [3].

Organizations such as the American Heart Association (AHA) provides basic CPR education through a “Family & Friends CPR Course” for participants from senior elementary school students to the elderly [4]. The World Health Organization’s (WHO) endorsement of “Kids Save Lives” has promoted the global implementation of CPR education for school children [5]. Although such education programs are useful, not all children achieve or maintain the recommended depth of chest compression of 5–6 cm [6]. Similar research have once again emphasized the importance of bystander CPR [7, 8]. To elevate bystander CPR education, the WHO and the European Resuscitation Council (ERC) have actively encouraged CPR education for school children. Children could get a positive attitude toward CPR education, and later they might enable their families and friends to learn CPR [9]. The AHA recommends that secondary school students could conduct good-quality compressions depth with least disruption [10]. In other words, when assessing good-quality chest compression and evaluating CPR education results, it is recommended to use a rate of predefined passing level that 5–6 cm depth of chest compression is achieved. The excellence of CPR was defined as an individual’s ability to achieve the desired compression depth; however, ascertaining a level could raise some educational challenges for young children. First, the target depth of 5–6 cm was reached by the most 14 years children [11]. Second, in an educational aspect, it is not right to accept or teach compression depth different from those recommended by the AHA or ERC. One solution to these issues involves applying learning theory: by slowly enhancing the proportion of appropriate CPR skills, participants may reach good-quality CPR skill levels in the course of time. These educational strategies require coordinated learning goals, taking into account the physical characteristics of different age groups [12]. Some scholars have noted particular strengths of school children: they constitute a significant proportion of the total population while retaining suitability for continuous and systematic CPR education [6, 13]. For these reasons, mandatory CPR education should exist in schools.

Korea has been developing its CPR education for school children. The Korea government introduced first aid education (including CPR) in the 1994 Emergency Medical Care Act. Also, CPR education was included in the health curriculum from fourth grade elementary school to the first grade of high school from 2009 in Korea [14]. According to the 2022 survey, 99% of high schools with health courses provided CPR education [15]. In another study, sixth grade children of elementary school had the highest confidence and propagation power in their CPR abilities as well as the highest overall skill scores, indicating that elementary school was an important time to start CPR education [16].

In Korea, CPR education is currently provided for children attending elementary school. However, not all elementary school students achieve or maintain the recommended chest compression depth of 5 cm. At this point, we had the following questions: Are Korean elementary school students performing chest compression at an adequate depth? What age can Korean elementary school students apply compression to an adequate depth? What weight, height or BMI allows Korean elementary school students to perform quality chest compression? Therefore, we conducted this study to verify the rates of performing chest compression at an adequate depth, the age for applying compression to an adequate depth, and appropriate weight, height or BMI for quality chest compressions among Korean elementary school students.

## Methods

### Study Subjects and Design

The total of 140 participants in third, fourth, fifth, and sixth grade students from an elementary school in Korea participated in this study. The school was gender-mixed, heterogeneous on the level of students’ socio-economic background, and CPR education was provided as a part of the school curriculum. A month before commencing the study, we visited a rural elementary school. With the principal’s permission, the authors visited classrooms in grades 3 through 6, explaining the study’s purpose and process. Two informed consent forms were distributed to interested students willing to participate. Those who completed the submission of informed consent, both for themselves and their parents, became the final subjects of this study. All participants and their parents provided informed consent before participating in the study. Children with physical handicaps or underlying diseases that significantly limit physical performance were excluded.

Prior to CPR education, all participants examined height, weight, and body mass index (BMI). And then, grip strength and back strength were measured. Next, CPR education was conducted by the certified instructor for 60 min, (Basic Course for General CPR, Korean Cardiopulmonary Resuscitation Association). Two paramedics with certificate of BLS instructor trained all children. They had an average teaching experience of 10 years in CPR education. The CPR procedure was based on the AHA 2015 guidelines.

Right after CPR education, same two paramedics assessed the CPR performance quality of every participant individually using Brayden Pro (Innosonian Inc., Seoul, Korea). To reduce influencing variance of the results, same researchers was responsible for the study.

Performance was recorded on a Brayden Pro manikin connected to a laptop running the PC-Skill Reporting-system version 2.21. The following variables were registered: chest compression depth, incomplete release, compression frequency and compressions with correct hand placement. The achieved level of excellence was defined in terms of the percentage of chest compressions with a depth of 5–6 cm.

### Statistical Analyses

Means of physical characteristics (height, weight, BMI, grip strength and back strength) and compression variables were reported per school grade. Means of physical characteristics and compression variables were reported based on the chest compression depth of 5 cm. T-test, and one-way ANOVA were carried out. Pearson’s correlation (r) was conducted to analyze the relationship between physical characteristics and quality of chest compression depth. A significance level was set at 0.05, two-tailed. Data analysis was performed using the SPSS/WIN 25.0 program.

## Results

### Baseline Characteristics of Subjects

As shown in Table 1, participants consisted of 140 students grade range 3–6 grade in elementary school. In height, weight, BMI, grip strength and back strength, differences according to school grade grew larger. The average height was 132.5 cm in the third grade, 137.5 cm in the fourth grade, 144.8 cm in the fifth grade, and 150.1 cm in the sixth grade. As school year increased, student height increased, and this was statistically significant (*p* = 0.000). Average weight was 30.4 kg in the third grade, 38.3 kg in the fourth grade, 42.4 kg in the fifth grade, and 49.3 kg in the sixth grade. As students’ grade level advanced, their weight increased, and there was a statistically significant difference (*p* = 0.000). BMI values were 17.2 in the third grade, 20.2 in the fourth grade, 20.0 in the fifth grade, and 21.6 in the sixth grade. As students advanced in their schooling, their BMI increased, and these values were statistically significant (*p* = 0.000).

**TABLE 1:** Baseline of subjects’ characteristics (South Korea, 2021).

Characteristics	Third grade (*n* = 35)	Fourth grade (*n* = 33)	Fifth grade (*n* = 35)	Sixth grade (*n* = 37)	t/F	*p*-value
Height (cm)	132.56 ± 6.71	137.56 ± 5.04	144.89 ± 7.52	150.12 ± 7.65	33.703	0.000 (a < c, a < d, b < c, b < d)
Weight (kg)	30.45 ± 7.05	38.34 ± 8.39	42.45 ± 11.22	49.34 ± 12.24	16.881	0.000 (a < b, a < c, a < d, b < d)
BMI	17.23 ± 2.65	20.23 ± 4.13	20.01 ± 3.87	21.67 ± 4.08	6.953	0.000 (a < b, a < d)
Grip strength (kg)	10.78 ± 2.23	13.34 ± 2.20	16.12 ± 3.94	18.90 ± 4.67	28.230	0.000 (a < c, a < d, b < c, b < d, c < d)
Back strength (kg)	27.12 ± 7.02	29.67 ± 6.71	33.45 ± 9.94	47.78 ± 15.06	20.875	0.000 (a < d, b < d, c < d

When there are differences between groups in post-hoc analysis, a marker to distinguish the groups. (a,b,c,d represents each group respectively).

The average grip strength was 10.7 kg in the third grade, 13.3 kg in the fourth grade, 16.1 kg in the fifth grade, and 18.9 kg in the sixth grade. As the students’ grade level went up, their grip strength increased, which was statistically significant (*p* = 0.000). The average back strength values were 27.1 kg in the third grade, 29.6 kg in the fourth grade, 33.4 kg in the fifth grade, and 47.7 kg in the sixth grade. Students’ back strength increased as they advanced in school years, which was statistically significant (*p* = 0.000).

### Accuracy of Compression Depth Right After CPR Education

Chest compression depth showed an average value lower than the guideline standard (5–6 cm) in all grades. It was 2.78 cm in the third grade, 3.25 cm in the fourth grade, 4.19 cm in the fifth grade, and 4.44 cm in the sixth grade, showing differences by grade. As the grade increased, the compression depth increased, which was statistically significant (*p* = 0.000). The chest compression depth accuracy was 2.9% in the third grade, 3.3% in the fourth grade, 21.6% in the fifth grade, and 30.6% in the sixth grade, showing differences by grade. As the grade increased, the compression depth accuracy increased, which was statistically significant (*p* = 0.000) (Table 2).

**TABLE 2:** Accuracy of compression depth right after CPR education (South Korea, 2021).

Characteristics	Third grade (*n* = 35)	Fourth grade (*n* = 33)	Fifth grade (*n* = 35)	Sixth grade (*n* = 37)	t/F	*p*-value
Compression depth (cm)	2.78 ± 0.62	3.25 ± 0.62	4.19 ± 0.65	4.44 ± 0.58	41.563	0.000*** (a < c, a < d, b < c, b < d)
Proper compression depth (%)	2.90 ± 13.23	3.34 ± 11.72	21.67 ± 32.44	30.67 ± 32.27	8.312	0.000*** (a < d, b < d)

*** *p* < 0.001.

When there are differences between groups in post-hoc analysis, a marker to distinguish the groups. (a,b,c,d represents each group respectively).

### Physical Condition for Excellent Chest Compression

Comparing student data for those who achieved the target level with those who did not also reveals some key findings. The average height of students reaching less than 5 cm of chest compressions was 139.9 cm, and the average height of those who reached 5 cm or more was 149.8 cm. As student height increased, their chest compression pressure depth increased, and this was statistically significant (*p* = 0.001). The average weight of successful participants was 53.6 kg while those who failed to reach the target averaged was 38.2 kg. As participant weight increased, chest compression pressure depth increased, and this was statistically significant (*p* = 0.000). The average BMI of participants who reached less than 5 cm of compression depth was 19.2, and the average BMI of those who exceeded 5 cm was 23.4. This was also significant to the pressure depth due to the increase in BMI (*p* = 0.001). The mean of grip strength and back strength were also significant different to the compression depth (Table 3).

**TABLE 3:** Characteristics based on compression depth of 5 cm (South Korea, 2021).

Characteristics	Under 5 cm	Over 5 cm	t/F	*p*-value
Height (cm)	139.93 ± 9.24	149.89 ± 8.06	3.146	0.001**
Weight (kg)	38.22 ± 11.36	53.69 ± 6.74	4.410	0.000***
BMI	19.24 ± 3.88	23.43 ± 2.15	3.505	0.001**
Grip strength (kg)	14.28 ± 4.39	19.78 ± 4.99	2.460	0.016*
Back strength (kg)	32.79 ± 11.69	46.63 ± 15.51	2.863	0.015*

* *p* < 0.05, **p < 0.01, ***p < 0.001.

### Factors Influencing Chest Compression Depth

To examine the factors influenced chest compression depth, multivariable regression analysis was conducted (Table 4). In multivariable regression analysis, the overall explanatory power of the regression model was significant at 57% (*R* ^2^ = 0.572, Adjusted *R* ^2^ = 0.545). According to the results of the regression analysis, it was observed that as the school grade increased, chest compression depth increased by 0.701 cm, and this was statistically significant (*p* < 0.001). Physical characteristics including weight were not significant.

**TABLE 4:** Regressions of compression depth (South Korea, 2021).

Characteristics	B	SE	β	t	*p*
Constant	−5.849	4.346		−1.346	0.181
Grade	0.701	0.082	0.866	8.537	0.000***
Height (cm)	0.048	0.032	0.499	1.517	0.133
Weight (kg)	0.101	0.053	1.315	1.915	0.058
BMI	0.197	0.108	0.848	1.831	0.970
Grip strength (kg)	−0.016	0.023	−0.080	−0.698	0.487
Back strength (kg)	0.000	0.007	0.005	0.052	0.959

*R* = 0.756, *R* ^2^ = 0.572, a*R* ^2^ = 0.545.

*** *p* < 0.001.

## Discussion

In Korea, CPR education is provided for children over fourth grade attending elementary school. However, as it was not sure that all elementary school students achieve or maintain the recommended chest compression depth of 5–6 cm, this study endeavored to confirm the rates of performing chest compression at an adequate depth, the age for applying compression to an adequate depth, and appropriate weight, height or BMI for quality chest compressions among Korean elementary school students.

Our study demonstrated that chest compression depth over 5 cm was dependent in school grade, height, weight, BMI, grip strength and back strength. There were the significant differences for all physical characteristics according to compression depth over 5 cm or not. Also there were positive correlations for all physical characteristics and chest compression depth over 5 cm, while weight was the strongest correlated with the compressions depth over 5 cm. This finding was in line with previous studies which showed that children’s physical characteristics played a major role in good-quality chest compressions [17–21]. In a previous study, elementary school students could improve their educational effectiveness and have more advantages in terms of cost efficiency and convenience of education; consequently [22]. Most of the existing studies have been conducted on adults, and there have been many other studies on medical personnel and related occupations [23, 24]. A previous study judged the quality of CPR reported that sixth grade students was higher in CPR skills than the fifth grade students [13]. Additionally, one researcher found that fifth and sixth grade students in elementary school are more effective than adults [6]. Some of previous studies mentioned that the proper chest compressions have begun in the fifth grade students, so fifth grade was appropriate for CPR education [13, 22, 25]. However, this study reported that chest compression depth in all grades was lower than standard guidelines of 5–6 cm.

Excellent chest compression depth between 5 and 6 cm was the major factor of good-quality CPR skill, as it was associated with survival outcome [26]. Nevertheless, there was a study that an analysis of the compression depth during CPR performed in the emergency room showed that the highest survival rate was found in a depth range of 4.3–5.53 cm (average, 4.56 cm) [7], giving an insight of CPR education for younger children. In our study, chest compression depth in all grades was lower than standard guidelines of 5–6 cm. But we found that as the school grade increased, the compression depth increased. Besides, mean compression depth of sixth grade students was 4.44 cm, indicating realistic possibility.

The subjects conducted adequate compressions depth over 5 cm were in a weight range of 46.95–60.44 kg (average, 53.69 kg). This study’s results were in line with other studies that have indicated that high-weight students showed much proper chest compression depth [11, 27]. And this correlation between weight and chest compression depth was reported among adults [12, 28–30]. In a prior study, the 50% of children weighed 50 kg around 13 years [31]. Flexion of the hip joint contributed to the entire power during CPR [31]. Therefore, a total weight of less than 53 kg did not limit a person’s ability to conduct sufficient compression, but might require far more effort than persons with larger weight. That’s why we mainly believed that age and physical characteristics could guide the customization of achievable chest compression depths. Therefore, training young children especially might require more effort and training while children weighting over 53 kg could achieve the target depth more easily.

Researches on proper age or school grade for starting CPR education have mostly focused on the physical condition to conduct good CPR skill. This study was the first study for the impact of physical strengths on the chest compression depth in children. Our result provided insight on physical strength dependent evolution of the CPR quality throughout the elementary school grades. We found that as the school grade increased, both hand grip strength and back strength increased. In addition, students with high-hand grip strength or high-back strength showed significantly much proper chest compression depth. The subjects conducted compressions depth over 5 cm had the hand grip strength of 19.78 kg and the back strength of 46.63 kg. Thus, it could be a strategy of CPR education for young children to grow children’ physical strength.

There were some researches on proper age for starting CPR education to conduct good CPR skill. One research reported that 12–14 years children showed good-quality CPR skill of 50% [32], another research mentioned good-quality CPR skill of 60% with 11–12 years children [33], and a previous research reported a good-quality CPR skill of 40% [34]. But our result showed that a depth of 5–6 cm was only reached by some of fifth and sixth grade, 21% and 30%, respectively. Some researches mentioned that 8–10 years children could start skills acquisition [6, 35]. In addition, there were several researches that far younger children could be successfully educated CPR [36–38]. Additionally, a prior study demonstrated progress in learning for individuals of all ages, irrespective of their initial age [14]. A recent investigation also revealed that schoolteachers are open to teaching CPR. They expressed agreement with the integration of first aid training into the curriculum for teachers pursuing teaching degrees [39]. This further supports the effectiveness of educational strategies for schoolchildren.

Several research reported that children with a higher weight were more probable in achieving higher excellent CPR skill. Maybe But, children were usually not educated to achieve a certain goal of good-quality CPR skill. One research reported that when educated for pre-defined 70% excellent level, adults were gradually able to achieve it [12]. So if instructors provided children with a particular good-quality CPR level, children could be more challenged and encouraged better performance with adequate chest compression depths. An emphasis on adequate compression depth might make children enhance chest compression depth. It should be needed to verify compression depth with children using a specific strategy such as ‘push as hard as you can’, contemplated age and physical characteristics.

Considering children who could physically not perform adequate chest compressions during CPR education, we suggested applying positive reinforcement differently concerning school grade. We should motivate elementary school students to push hard for better results in CPR education, focusing on the depth of chest compression to achieve good-quality CPR.

There are some limitations in this study. This study was an observational study and used small study population. Also the participation in one school or monitoring in another might have influenced the results. Thus those limit the generalizability of our results. In addition, we did not particularly emphasize the achievement of good-quality CPR skill during CPR education. Therefore, the results of this study would be underestimated. Besides, we could not consider the exertion, which would actually be an additional restriction to keep a constant good-quality CPR skill during CPR, because this results were based on a five-cycle CPR examination.

### Conclusion

The proportion of Korean elementary school students performing chest compressions at an appropriate depth was low. The grade level had a significant impact on achieving an appropriate depth of chest compressions among elementary school students. Physical factors such as height, weight, BMI, grip strength, and back strength did not show significant correlations. Therefore, it appears challenging for elementary school students to achieve an appropriate depth of chest compressions through CPR education and practice. Thus, a strategy emphasizing the importance of sufficient chest compressions during CPR education, particularly targeting elementary school students, seems necessary to encourage greater effort.
